# Genetic testing for misclassified monogenic diabetes in Māori and Pacific peoples in Aōtearoa New Zealand with early-onset type 2 diabetes

**DOI:** 10.3389/fendo.2023.1174699

**Published:** 2023-05-10

**Authors:** Zanetta Toomata, Megan Leask, Mohanraj Krishnan, Murray Cadzow, Nicola Dalbeth, Lisa K. Stamp, Janak de Zoysa, Tony Merriman, Phillip Wilcox, Ofa Dewes, Rinki Murphy

**Affiliations:** ^1^ Department of Medicine, Waipapa Taumata Rau, The University of Auckland, Auckland, New Zealand; ^2^ Maurice Wilkins Centre for Molecular Biodiscovery, Auckland, New Zealand; ^3^ Department of Biochemistry, University of Otago, Dunedin, New Zealand; ^4^ Division of Clinical Immunology and Rheumatology, University of Alabama at Birmingham, Birmingham, AL, United States; ^5^ Department of Biostatistics, Graduate School of Public Health, University of Pittsburgh, Pittsburgh, Pittsburgh, PA, United States; ^6^ Department of Medicine, University of Otago, Christchurch, Christchurch, New Zealand; ^7^ Department of Mathematics and Statistics, University of Otago, Dunedin, New Zealand; ^8^ Langimalie Research Centre, Auckland, New Zealand; ^9^ Centre of Methods and Policy Application in the Social Sciences, The University of Auckland, Auckland, New Zealand

**Keywords:** precision medicine, monogenic diabetes, type 2 diabetes, molecular diagnosis, maturity-onset diabetes of the young (MODY), maternally inherited diabetes and deafness (MIDD), Aotearoa (New Zealand), Polynesian

## Abstract

**Aims:**

Monogenic diabetes accounts for 1-2% of diabetes cases yet is often misdiagnosed as type 2 diabetes. The aim of this study was to examine in Māori and Pacific adults clinically diagnosed with type 2 diabetes within 40 years of age, (a) the prevalence of monogenic diabetes in this population (b) the prevalence of beta-cell autoantibodies and (c) the pre-test probability of monogenic diabetes.

**Methods:**

Targeted sequencing data of 38 known monogenic diabetes genes was analyzed in 199 Māori and Pacific peoples with BMI of 37.9 ± 8.6 kg/m^2^ who had been diagnosed with type 2 diabetes between 3 and 40 years of age. A triple-screen combined autoantibody assay was used to test for GAD, IA-2, and ZnT8. MODY probability calculator score was generated in those with sufficient clinical information (55/199).

**Results:**

No genetic variants curated as likely pathogenic or pathogenic were found. One individual (1/199) tested positive for GAD/IA-2/ZnT8 antibodies. The pre-test probability of monogenic diabetes was calculated in 55 individuals with 17/55 (31%) scoring above the 20% threshold considered for diagnostic testing referral.

**Discussion:**

Our findings suggest that monogenic diabetes is rare in Māori and Pacific people with clinical age, and the MODY probability calculator likely overestimates the likelihood of a monogenic cause for diabetes in this population.

## 1 Introduction

Monogenic diabetes is caused by single gene mutations (or rarely by deletions) and accounts for an estimated 1-2% of diabetes cases in major Western populations (1–4). These diabetes subtypes are important to diagnose correctly, as they have distinct therapeutic implications compared to more common polygenic forms of type 1 (T1D) and type 2 diabetes (T2D), with which they are frequently misclassified. Correct identification of monogenic diabetes is challenging as it relies on clinicians detecting subtle differentiating clinical features that are atypical for either type 1 (e.g., absence of antibodies, persisting C-peptide) or type 2 diabetes (e.g., absence of obesity, lack of clinical insulin resistance) before requesting a diagnostic genetic test (5). In the presence of increasing obesity, and younger presentation of T2D, such clinical cues are less informative. The historic label for monogenic diabetes was ‘Maturity-Onset Diabetes of the Young’ (MODY), with the classic triad of diabetes diagnosed under 25 years of age, with autosomal dominant family history of diabetes, and without insulin requirements. This triad does not differentiate from the growing population of young-adult onset of T2D, hence a tool often used by clinicians, known as the MODY probability calculator (MPC), is used to calculate the pre-genetic-test probability (PTP) of testing positive for monogenic diabetes using a combination of broad clinical criteria (i.e., age at diabetes diagnosis, time to insulin treatment, BMI, HbA1c and family history of diabetes) (6).

In Aōtearoa New Zealand (NZ), Māori and Pacific peoples have a prevalence of diabetes that is 7.9% and 13.6%, respectively (7). It is likely there is inadequate discriminatory clinical criteria for monogenic diabetes in these populations, given the overlapping clinical features of monogenic diabetes with T2D, both occurring at a similar young age group in the presence of family history of diabetes, and with similar long duration before time to insulin treatment (5, 8). In this study, we aimed to investigate whether misclassification of monogenic diabetes occurs in Māori and Pacific patients with clinically classified T2D within 40 years of age, their PTP estimated using the MPC, and their prevalence of beta-cell autoantibodies characteristic of T1D. We used phenotype and targeted genome sequence data from Māori and Pacific participants of the Genetics of Gout, Diabetes, and Kidney disease (GoGDK) study (9).

## 2 Materials and methods

### 2.1 Participants

All participants provided written informed consent for the collection of their samples and subsequent analyses. The ethical approval for this study was given by the New Zealand Multi-Region Ethics Committee (MEC/05/10/130; MEC/10/09/092; MEC/11/04/036). 567 participants who identify as Māori and/or Pacific (Polynesian) from the GoGDK study of Aōtearoa/New Zealand were included for targeted sequencing of their DNA samples on the basis of T2D diagnosed within 40 years of age (n=199 cases) or age-matched controls not having T2D (n=368) (9). The T2D ascertainment of this cohort has been described previously (10). A range of clinical, demographic, and biochemical measurements were collected from these participants. Stored serum samples from participants with T2D were tested for the presence of serum autoantibodies GAD (glutamic decarboxylase), IA-2 (islet antigen 2), and ZnT8 (zinc transporter 8) using the 3-screen islet cell autoantibody Enzyme-Linked Immunosorbent Assay (ELISA) kit (RSR limited, Cardiff, UK) for combined quantitative determination of GAD, IA-2 and ZnT8 autoantibodies.

### 2.2 MODY probability calculator

We examined the MPC PTPs in screening this cohort of Māori and Pacific peoples for MODY monogenic diabetes as developed by Shields et al. (6). The model considers the following variables: current age (years), age at diabetes diagnosis (years), gender, the historical or ongoing use of insulin and/or orally administered anti-hyperglycemic agents (OHAs), time to insulin treatment (where applicable), BMI (kg/m^2^), HbA1c (%), and a presence of a family history of diabetes. We used the available clinical information required to calculate the PTPs for GoGDK participants with clinically classified, young-adult onset T2D. An MPC PTP > 20% threshold was used in accordance with NZ’s national testing guidance from the New Zealand Society for the Study of Diabetes (NZSSD).

### 2.3 Targeted sequencing

We performed targeted sequencing of the exons and the 5’ and 3’ UTR regions of 38 genes associated with monogenic diabetes, syndromic forms of diabetes, lipodystrophy, and hyperinsulinemia. This included the 13 genes known or predicted to cause MODY at the time the gene panel was generated (*APPL1* [HGNC:24035] not on the gene panel). The full list of 38 genes analyzed for this study are in **Supplementary Table 1**. In addition, the mitochondrial genetic variant, m.3243A>G, in the gene *MT-TL1* (HGNC:7490) was examined by TaqMan real-time PCR in accordance with the method described previously (11).

Sequencing was performed at the McDonnell Genome Institute at Washington University in St. Louis, Missouri, using an Illumina HiSeq 2000 system and a total target capture of 5Mb. DNA libraries were bar coded using the Illumina index read strategy. A Snakemake workflow was used to process the sequencing data. Aligned BAM files provided by the sequencing facility were pre-processed into leavened FASTQ files using Samtools (v1.10). Sequence reads from the FASTQ files were aligned to GRCh37/hg19 using the Burrows-Wheeler algorithm (BWA; v 0.7.17) (12). Using Picard software^1^ and the Genome Analysis Toolkit (GATK; v4.1.7), PCR duplicate reads were marked and base quality score recalibration and indel realignment were conducted. GATK HaplotypeCaller (v4.1.7) was used to call variants, such as single nucleotide polymorphisms (SNPs) and small indels. All variants were annotated using SnpEff (v5.1) (13), and the Genome Aggregation Database (gnomAD v2.1.1^2^). Multi-allelic and monomorphic variants, along with SNPs within 10 base pairs of an indel were filtered from analysis. Additionally, only variants with a genotype quality score >= 20 (GQ >= 20) and a filtered read depth > 10 (DP > 10) were used for further analysis.

### 2.4 Variant annotation

Pathogenicity of variants were classified according to the ClinGen Monogenic Diabetes Expert Panel Specifications to the ACMG/AMP guidelines (14, 15). The guidelines classify genetic variants into 5 different categories: (B) benign, (LB) likely benign, (VUS) variant of uncertain significance, (LP) likely pathogenic, or (P) pathogenic. The variants were annotated using the transcripts from the Matched Annotation from the NCBI and EBI (MANE) project, an effort that harmonizes RefSeq and Ensembl transcripts (16). The list of MANE transcripts used for each monogenic diabetes gene is shown in **Supplementary Table 1**. The focus was on coding variants that could alter the protein sequence, such as protein-truncating variants (i.e., nonsense, frameshift, or splice-site variants that abolish a canonical splice-site [-2 or +2 bases from exon boundary]) and missense variants. Missense variants were further annotated using the REVEL score (0-1 range), which is a tool that considers in aggregate a combination of 13 individual *in silico* prediction tools (SIFT, PolyPhen2-HDIV, PolyPhen2-HVAR, LRT, MutationTaster, MutationAssessor, FATHMM, PROVEAN, MetSVM, MetLR, CADD, FATHMM-MKL, and fitCons) to suggest pathogenic or benign impact. A REVEL score >= 0.70 was used as supportive evidence of pathogenicity for the ACMG/AMP PP3 criterion. Variants with a minor allele frequency (MAF) greater than or equal to 0.01% (MAF >= 0.0001) in the population reference databases gnomAD, ExAC (Exome Aggregation Consortium), 1000 Genomes Project, and ESP (Exome Sequencing Project), were filtered out from further analysis (ClinGen ACMG/AMP BA1 stand-alone criterion (17)). Additionally, as there remains a lack of representation of Māori and Pacific peoples in these international databases, we filtered out variants with MAF > 0.01% in the 368 Māori and Pacific controls without diabetes.

## 3 Results

### 3.1 Participant clinical characteristics

**Figure 1:**
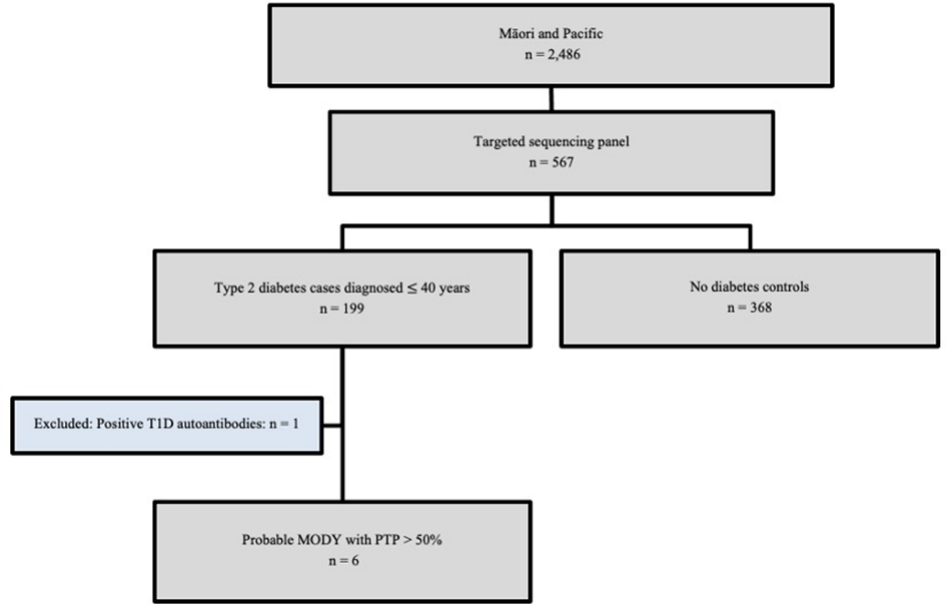
GoGDK CONSORT diagram of Māori and Pacific participants in the GoGDK study present on the sequencing panel. 567 participants who identified as Maāori and/or Pacific from the GoGDK study of Aōtearoa/New Zealand (n=567) were included for targeted genome sequencing, such that 199 cases were diagnosed with T2D within 40 years of age, and 368 were age-matched controls without diabetes. T1D, type 1 diabetes; MODY, maturity-onset diabetes of the young, PTP, pre-test probability.

A total of 199 Māori and Pacific participants with T2D at the time of recruitment underwent targeted sequencing, with one individual removed from further analysis having tested positive for the presence of T1D autoantibodies GAD/IA-2/ZnT8 (**Figure 1**). This individual had an HbA1c of 97 mmol/mol (11%) at the time of recruitment at age 53 and had been diagnosed with diabetes at 32 years of age. Their time to insulin was 16 years after their initial T2D diagnosis. This left 198 participants that were evaluated for a monogenic diabetes cause. There were 368 Māori and Pacific control participants without diabetes as proxy for general population MAFs to aid variant curation for monogenic diabetes. The mean age for cases with T2D at the time of recruitment was 45.14 ± 12.12 years, with the age at diabetes diagnosis ranging from 3-40 years (**Supplementary Figure 1**). The mean age at time of recruitment was 44.07 ± 15.56 years for controls. The mean BMI at time of recruitment was 35.5 ± 12.6 kg/m^2^ for controls and 37.80 ± 8.56 kg/m^2^ for cases with T2D. A summary of other baseline characteristics, such as triglycerides, total cholesterol, status of gout and chronic kidney disease for both case and control groups are listed in **Table 1**.

**Table 1:** Clinical characteristics of GoGDK participants on the sequencing panel.

Characteristic	N available	Cases Mean ± SD	N available	Controls Mean ± SD
**N**	199		368	
**Age (years)**	199	45.14 ± 12.12	368	44.07 ± 15.56
**Female (n(%))**	199	117 (58.8%)	368	152 (41.3%)
**Weight (kg)**	189	107.67 ± 29.41	346	104.4 ± 38.1
**Height (cm)**	183	169.37 ± 9.92	345	171 ± 9.4
**BMI (kg/m^2^)**	191	37.80 ± 8.56	368	35.5 ± 12.6
**Serum urate (mmol/L)**	185	0.34 ± 0.14	322	0.4 ± 0.1
**eGFR (ml/min/1.73/m^2^)**	173	58.16 ± 34.37	185	80.4 ± 27.6
**Total cholesterol**	175	4.68 ± 1.46	185	4.8 ± 1.0
**Triglycerides (mmol/L)**	175	3.42 ± 5.02	186	1.9 ± 1.2
**HDL (mmol/L)**	178	1.06 ± 0.32	313	1.2 ± 0.4
**LDL (mmol/L)**	145	2.34 ± 0.85	177	2.7 ± 0.8
**Gout (n(proportion))**	197	32 (0.162)	356	135 (0.379)
**Chronic kidney disease (n(proportion))**	174	65 (0.374)	205	10 (0.049)

### 3.2 Monogenic diabetes gene variants

None of the Māori and Pacific T2D cases (0/199) from the GoGDK study had a P/LP variant in any of the 13 MODY genes. Additionally, while we found variants (n=81) in the other 25 non-MODY genes in this study, none of these were determined P/LP for monogenic diabetes or other syndromes.

### 3.3 MODY probability score

**Figure 2:**
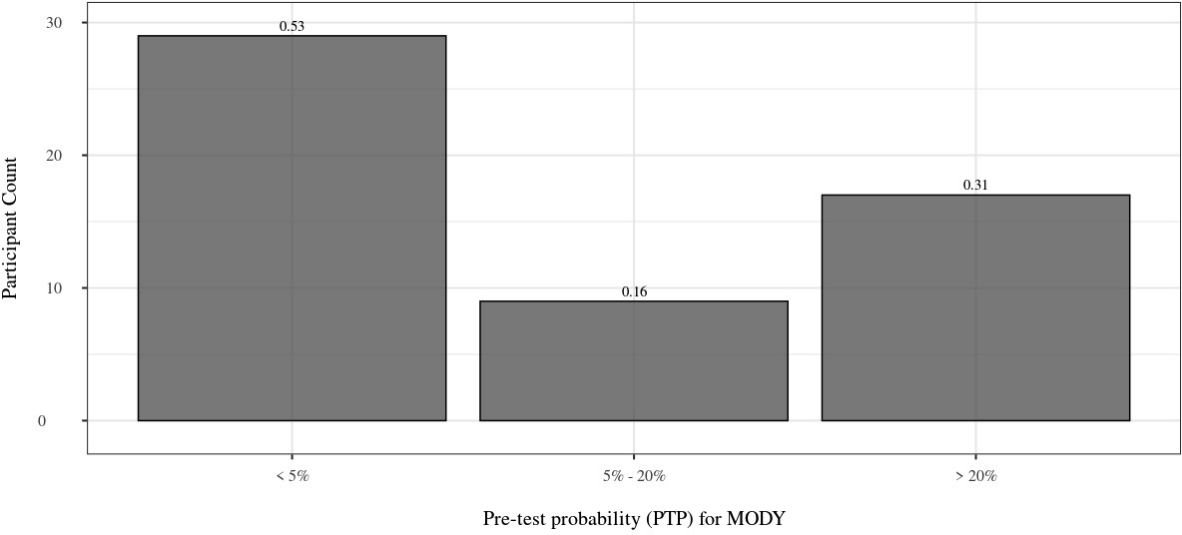
Distribution of PTP. Distribution of PTP for the 55 Māori and Pacific participants with full clinical information available. 53% of participants have a PTP < 5%, whereas 31% have a PTP > 20%. The proportion of individuals within each group is above each bar. PTP, pre-test probability; MODY, maturity onset diabetes of the young.

In total, there were 55 Māori and Pacific participants on the sequencing panel with sufficient clinical information to calculate PTPs for MODY monogenic diabetes. Of this group, 36.4% (n=20) were female. The mean age at time of recruitment was 51.6 ± 12.3 years, and the mean BMI was 34.7 ± 7.2 kg/m^2^. 42 participants (42/55 = 76.4%) had at least one parent affected by diabetes, with 2 participants (2/24 = 16.7%) having both parents and a sibling with diabetes. The participants were treated with OHAs (n=18; 18/49 = 36.7%) and insulin (n=36; 36/49 = 73.5%) (**Table 2**). The participants had a mean HbA1c of 8.9 ± 2.6% (74 mmol/mol ± 5 mmol/mol). There were 30.9% (n=17) of participants that had a PTP > 20%, while 52.7% (n=29) had a PTP < 5%. The PTP ranged from 4.60% to 75.49% (**Figure 2**).

**Table 2:** Clinical characteristics of the 55 GoGDK participants with PTP for MODY.

Characteristic	N available	Mean ± SD (min - max)
**Age (years)**	55	51.6 ± 12.3 (20 - 78)
**Female (n(%))**	55	20 (36.4)
**BMI (kg/m^2^)**	55	34.7 ± 7.2 (21.1 - 52.44)
**Diabetes onset age (years)**	55	27 ± 8 (11 – 40)
**HbA1c (%)**	55	8.9 ± 2.6 (4.4 – 14.9)
**Family history of diabetes (n(%))**	55	44 (86.4)
**Family members with diabetes (n(%))**	55	48 (87.3)
0 – 1	55	20 (36.4)
> 2	55	35 (63.6)
Family members with diabetes (n(%))		
Mother (M)	47	25 (53.2)
Father (F)	42	23 (54.8)
Siblings (S)	34	28 (82.4)
(M/F + S)	34	26 (76.5)
M + F	36	6 (16.7)
Other combinations (Grandparent or child)	35	16 (45.7)
Therapy (n(%))		
Insulin	49	36 (73.5)
OHA	49	18 (72)
OHA + Insulin	49	12 (24.5)

SD, standard deviation; BMI, body mass index; HbA1c, hemoglobin A1c, OHA, oral hypoglycemic agents.

## 4 Discussion

Our study utilized a targeted sequencing approach that included a total of 38 genes associated with monogenic diabetes, syndromic forms of diabetes, lipodystrophy, and insulin resistance syndromes. It was hypothesised that in this sample set of early-onset T2D cases diagnosed within 40 years of age, there would be an estimated 2-4 misclassified monogenic diabetes cases, assuming a background monogenic diabetes prevalence like the 1-2% estimation ascertained in European cohorts (1–4, 18, 19). However, no cases of misclassified monogenic diabetes were detected in this sample of 199 Māori and Pacific people clinically diagnosed with T2D between 3-40 years of age. The single individual with beta-cell autoantibodies represents misclassified latent autoimmune diabetes of adults (LADA) or T1D. Given their time to insulin was 16 years after their initial diabetes diagnosis, this suggests LADA. T1D generally results in diabetic ketoacidosis presentations necessitating insulin treatment within 3 years after diabetes diagnosis (20). Poor glycaemic control in this individual indicates the problem of clinical inertia because earlier initiation of insulin should have occurred as part of optimal diabetes therapy, which may have been more successful if the correct diagnosis of LADA had been made.

It has been suggested that *HNF1A*/*HNF4A*-MODY can present at up to 45 years of age and should be included as an age cut-off to consider all possible cases of this MODY subtype (21). However, given the decreasing incidence of later presentation of monogenic diabetes combined with the marked increase in incidence of T2D, it is unlikely that we would have missed monogenic diabetes cases from the GoGDK cohort diagnosed between 35-45 years of age.

Furthermore, among the 55 individuals on the sequencing panel that were diagnosed with T2D within 40 years of age and had their full clinical information to calculate their PTPs for testing positive for MODY, 17 participants (30.9%) had a PTP > 20%, suggesting their referral for genetic testing (**Table 2**). The confirmed absence of T1D autoantibodies in those participants increased the possibility of true T2D or monogenic diabetes. Despite this, we did not identify a monogenic cause in any of the 17 individuals, thus signifying a lowered specificity for the MPC to identify MODY monogenic diabetes in our study of Māori and Pacific individuals with clinically classified T2D. A couple limitations to this study include the limited information to calculate PTPs for all individuals on the sequencing panel, and the inability to comment on the sensitivity of the MPC in the Māori and Pacific population, as we did not examine confirmed cases of monogenic diabetes. It should be recognized that a possible explanation for a low proportion of monogenic diabetes in the Māori and Pacific populations of NZ could be due to factors underlying reduced referrals and diagnostic testing. A 2023 audit of referrals for monogenic diabetes genetic testing in NZ showed that for Māori patients, the number of referrals correlated with the Māori diabetes prevalence in NZ (15%), but Pacific patients had both low referral (8%) and detection rates (5%) of monogenic diabetes (22).

The MPC assumes a MODY prevalence of 4.6% for patients with an initial diagnosis of T2D, as derived from the United Kingdom (UK) population (23). More specifically, the MPC considers a UK prevalence of all diabetes cases diagnosed below 35 years of age to be 26% due to T2D, and with 1.2% comprised of MODY diabetes cases that did not receive insulin treatment within 6-months of their diabetes diagnosis, thus fitting the “Type 2” criteria (1.19/26 = 0.046) (23). This concords with the TODAY study which found 4.5% of monogenic diabetes (22/488 cases) in a USA population of youth (10-17 years of age) diagnosed with T2D (24). The prevalence of diabetes in NZ is age-related and estimated as affecting 10% and 6% of the adult Pacific and Māori population by the age of 40 years (25). The incidence of T2D in Māori and Pacific children in NZ (<15 years of age) has been estimated as 4.1/100,000 and 5.9/100,000, respectively (26). Therefore, similar to what was seen in other non-European populations with a higher prevalence of young-onset T2D (27, 28), Māori and Pacific participants with early-onset diabetes would have an inflated risk of monogenic diabetes by this UK-derived prediction model.

Importantly, we also examined variants in genes that are associated with syndromic monogenic diabetes (e.g., m.3243A>G mutation in the *MT-TL1* mitochondrial gene, and *WFS1*), as these have been shown to be relatively common, and individuals would present overlapping diabetes phenotypes with non-syndromic monogenic diabetes (29, 30). In summary, it is likely that the underlying assumption of a 4.6% background prevalence of MODY by the MPC is an overestimate of the prevalence of MODY within our subset of Māori and Pacific participants (0/199). However, we acknowledge the limited sample size, hence the prevalence of MODY in larger cohorts of Māori and Pacific participants with early-onset T2D still requires further investigation.

## Data availability statement

The datasets presented in this article are not readily available because of consent restrictions. Requests to access the datasets should be directed to the corresponding authors, ZT (zanetta.toomata@auckland.ac.nz) and RM (r.murphy@auckland.ac.nz).

## Ethics statement

The studies involving human participants were reviewed and approved by New Zealand Multi-Region Ethics Committee. The patients/participants provided their written informed consent to participate in this study (MEC/05/10/130; MEC/10/09/092; MEC/11/04/ 036).

## Author contributions

RM conceived and designed the study. ZT drafted the manuscript and conducted the analyses. MC assisted with the data processing. MK contributed to gene variant curation. ML designed the sequencing panel with input from RM and TM. ND, LS, JD, RM and TM were responsible for oversite of the GOGKD study. PW, OD, and RM supervised ZT. All authors have edited, reviewed and approved the paper. All authors contributed to the article and approved the submitted version.

## Footnotes

1
https://broadinstitute.github.io/picard/

2
https://gnomad.broadinstitute.org
