# Supplementary material for: Genetic testing for misclassified monogenic diabetes in Māori and Pacific peoples in Aōtearoa New Zealand with early-onset type 2 diabetes
Source: Front Endocrinol (Lausanne). 2023 May 10;14:1174699. doi: 10.3389/fendo.2023.1174699 (PMC10206310; doi:10.3389/fendo.2023.1174699)
Supplement: Supplementary file 1 [file DataSheet_1.pdf]

Supplementary Material

Genetic testing for misclassified monogenic diabetes in Māori and Pacific peoples in Aōtearoa New Zealand with early-onset type 2 diabetes

Zanetta Toomata*, Megan Leask[3,4], Mohanraj Krishnan[5], Murray Cadzow[3], Nicola Dalbeth[1], Lisa K Stamp[6], Janak de Zoysa[1], Tony Merriman[3,4], Phillip Wilcox[2,7], Ofa Dewes[2,8,9], Rinki Murphy*[1,2]

*** Co-correspondence:** Zanetta Toomata: zanetta.toomata@auckland.ac.nz; Rinki Murphy: r.murphy@auckland.ac.nz

1 Supplementary Figures and Tables

1.1 Supplementary Figures

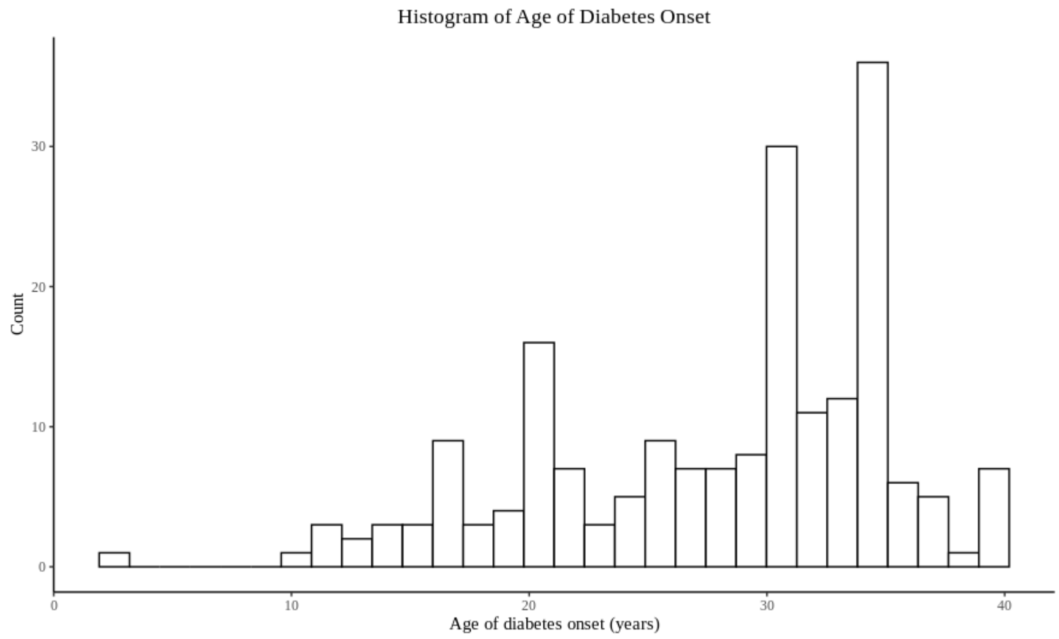

Supplementary Figure 1. The distribution of the age of type 2 diabetes onset for the 199 type 2 diabetes cases in the GoGDK. The age of type 2 diabetes onset ranged from 3-40 years of age.

1.2 Supplementary Tables

Supplementary Table 1. Gene panel list

Gene	OMIM ID	MANE transcript
ABCC8	600509	NM_000352.6
AGPAT2	603100	NM_006412.4
BLK	191305	NM_001715.3
BSCL2	606158	NM_001122955.4
CAV1	601047	NM_001753.5
CEL	114840	NM_001807.6
CISD2	611507	NM_001008388.5
EIF2AK3	604032	NM_004836.7
GATA4	600576	NM_001308093.3
GATA6	601656	NM_005257.6
GCK	138079	NM_000162.5
GLIS3	610192	NM_001042413.2
GLUD1	138130	NM_005271.5
HADH	601609	NM_005327.7
HNF1A	142410	NM_000545.8
HNF4A	600281	NM_175914.5
HNF1B	189907	NM_000458.4
INS	176730	NM_000207.3
INSR	147670	NM_000208.4
KCNJ11	600937	NM_000525.4
KLF11	603301	NM_003597.5
LMNA	150330	NM_170707.4
NEUROD1	601724	NM_002500.5
PAX4	167413	NM_001366110.1
PAX6	607108	NM_001368894.2
PDX1	60073	NM_000209.4
PLIN1	170290	NM_002666.5
POLD1	174761	NM_002691.4
PPARG	601487	NM_138711.6
PPP1R3A	600917	NM_002711.4
PTF1A	607194	NM_178161.3
PTRF	603198	NM_012232.6
RFX6	612659	NM_173560.4
SIM1	603128	NM_005068.3
SLC19A2	603941	NM_006996.3
SLC2A2	138160	NM_000340.2
WFS1	606201	NM_006005.3
ZFP57	612192	NM_001109809.5
